# Relação entre Ativação Endotelial e Índice de Estresse e Ectasia da Artéria Coronariana

**DOI:** 10.36660/abc.20260242

**Published:** 2026-09-21

**Authors:** Cihan Aydin, Hüseyin Orta, Aykut Demirkiran, Nadir Emlek

**Affiliations:** 1 Department of Cardiology Faculty of Medicine Tekirdag Namık Kemal University Tekirdag Turquia Department of Cardiology, Faculty of Medicine, Tekirdag Namık Kemal University, Tekirdag – Turquia; 2 Department of Cardiology Faculty of Medicine Recep Tayyip Erdoğan University Rize Turquia Department of Cardiology, Department of Cardiology, Faculty of Medicine Recep Tayyip Erdoğan University, Rize – Turquia

**Keywords:** Endotélio Vascular, Dilatação Patológica, Biomarcadores

## Abstract

**Fundamento:**

A ectasia da artéria coronariana (EAC) é caracterizada pela dilatação anormal das artérias coronárias e está associada à disfunção endotelial e à inflamação sistêmica. O Índice de Ativação e Estresse Endotelial (EASIX) surgiu como um novo biomarcador que reflete a ativação endotelial e o estresse inflamatório.

**Objetivos:**

Este estudo teve como objetivo avaliar a associação entre a pontuação EASIX e a presença de EAC.

**Métodos:**

Um total de 300 pacientes submetidos a angiografia coronária. Os dados coletados entre julho de 2018 e dezembro de 2025 foram analisados retrospectivamente. O estudo foi delineado como um estudo caso-controle. Os pacientes foram divididos em dois grupos de acordo com a presença de EAC (150 pacientes com EAC e 150 controles). O escore EASIX foi calculado utilizando a seguinte fórmula: EASIX=LDH × Creatinina/Plaquetas . A análise da curva ROC ( *Receiver Operating Characteristic* ) foi realizada para determinar o valor preditivo do EASIX para EAC. A análise estatística do índice de Youden foi realizada para avaliar o poder diagnóstico da análise ROC e calcular o valor de corte ideal. Um valor de p < 0,05 foi considerado estatisticamente significativo.

**Resultados:**

Os pacientes com EAC apresentaram pontuação EASIX significativamente maior em comparação com os controles (1,46 ± 0,33 vs. 0,98 ± 0,74). Na análise ROC, o escore EASIX foi de 72% sensibilidade e 58% especificidade quando o valor de corte para EASIX ≥0,74 (AUC=0,688, IC 95%:0,618-0,759, p<0,001). Na análise de regressão logística multivariável, o escore EASIX foi identificado como um preditor independente de EAC (OR = 2,12, IC 95%: 1,35–3,34, p = 0,001).

**Conclusão:**

O escore EASIX foi associado de forma independente à presença de EAC e demonstrou capacidade discriminativa moderada.

## Graphical abstract

**Figura Central:**
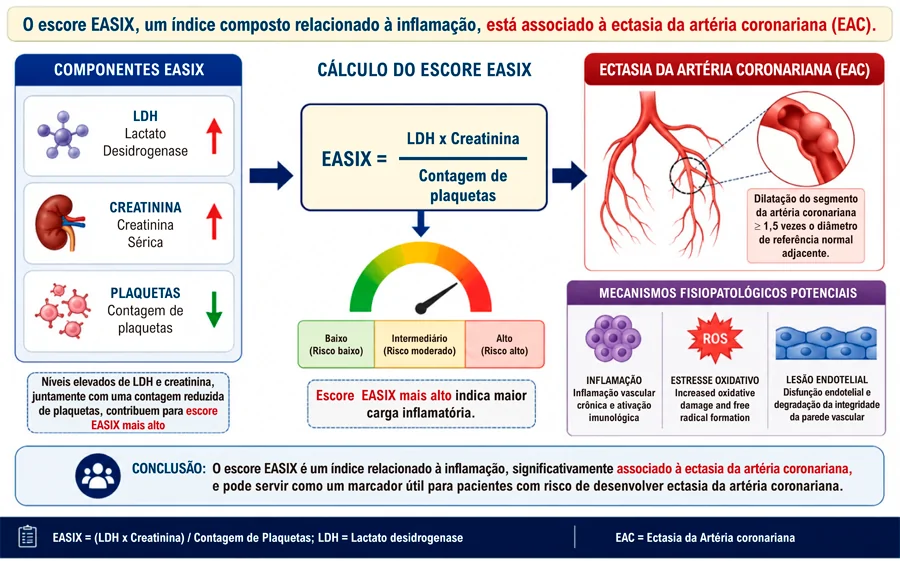
: Relação entre Ativação Endotelial e Índice de Estresse e Ectasia da Artéria Coronariana

## Introdução

A ectasia da artéria coronariana (EAC) é definida como uma dilatação localizada ou difusa de uma artéria coronariana que excede 1,5 vezes o diâmetro dos segmentos normais adjacentes.^1^ Ectasia se refere a dilatações da artéria coronariana de até 1,5 vezes, enquanto aneurismas se referem a dilatações superiores a duas vezes.^2^ A EAC pode ser adquirida ou congênita. Apesar de ser frequentemente considerada uma variação da doença arterial coronariana (DAC), a EAC é uma entidade clínica distinta, com características hemodinâmicas e fisiopatológicas diferentes. A importância clínica da EAC na prática cardiológica de rotina é demonstrada por relatos de que sua incidência em pacientes submetidos a angiografia coronária varia de 1% a 5%.^3^

A aterosclerose é responsável por 50% da patogênese, enquanto síndromes congênitas como a síndrome de Kawasaki, a síndrome de Ehlers-Danlos, a síndrome de Marfan e a doença de Takayasu, ou doenças inflamatórias do tecido conjuntivo como esclerodermia, poliarterite nodosa e lúpus eritematoso sistêmico também estão envolvidas.^3^ Esses diversos fatores etiológicos implicam que a EAC é uma doença vascular complexa que envolve várias vias biológicas, e não apenas uma anomalia morfológica.

Embora a fisiopatologia exata permaneça obscura, inflamação, disfunção endotelial e degradação da matriz extracelular são considerados mecanismos principais. Especificamente, a ativação excessiva de enzimas proteolíticas, como as metaloproteinases da matriz, pode danificar a parede arterial e causar dilatação inadequada. A disfunção endotelial é um fator chave no desenvolvimento de problemas vasculares. Ela aumenta a trombogenicidade, prejudica a regulação do tônus vascular e modifica a dinâmica do fluxo sanguíneo coronário, fatores que podem colocar os pacientes em risco de eventos cardiovasculares adversos.

Recentemente, o Índice de Ativação e Estresse Endotelial (EASIX), calculado a partir de exames laboratoriais padrão, foi introduzido como uma nova forma de mensurar danos endoteliais e estresse inflamatório. O EASIX foi inicialmente utilizado em contextos hematológicos, mas desde então demonstrou-se estar associado a um prognóstico desfavorável em diversas doenças sistêmicas e cardiovasculares. A lactato desidrogenase (LDH), a creatinina e as plaquetas (PLT) estão incluídas no EASIX, que foi recentemente desenvolvido e validado para mensurar a extensão do dano endotelial após o transplante alogênico de células-tronco.^4^ A relação entre o EASIX e diversos marcadores de ativação endotelial foi elucidada: supressor de tumorigenicidade-2, interleucina-18 (IL-18), ligante 8 da quimiocina-XC (CXCL8) e fator de crescimento semelhante à insulina-1.^5^ A LDH, a creatinina e a contagem de PLT, em conjunto, representam danos teciduais, função renal e equilíbrio hemostático, todos intimamente relacionados à integridade endotelial.^4 , 6^ Além disso, em diversas populações, incluindo pacientes com COVID-19, mieloma múltiplo, câncer de pulmão de pequenas células, linfoma difuso de grandes células B, sepse e DAC, o EASIX é um indicador válido de disfunção endotelial e risco de mortalidade.^7 - 10^

A escore EASIX tem sido associado a desfechos adversos em diversas doenças cardiovasculares e sistêmicas.^6^ Vários estudos demonstraram sua utilidade na predição de mortalidade entre pacientes com DAC, infarto agudo do miocárdio, acidente vascular cerebral (AVC) e hipertensão.^4 , 6^No entanto, os dados existentes sobre a associação entre o escore EASIX e a EAC são limitados e inconclusivos.

Portanto, este estudo teve como objetivo avaliar a associação entre o escore EASIX e a presença de EAC, bem como explorar a potencial relevância clínica do EASIX nessa população.

## Métodos

### População do estudo

Entre julho de 2018 e dezembro de 2025, 150 pacientes submetidos a angiografia em nossa clínica e diagnosticados com EAC, e 150 pacientes com artérias coronárias normais detectadas durante o procedimento de angiografia, foram retrospectivamente e aleatoriamente incluídos no estudo como grupo controle. Nosso estudo foi concebido como um estudo retrospectivo de caso-controle.

A EAC foi definida como o lúmen da artéria coronariana excedendo o diâmetro máximo de um vaso normal adjacente em mais de 1,5 vezes.^1^ Oclusão significativa e DAC foram definidas como estenose da artéria coronariana superior a 50%. Pacientes com doença sistêmica grave, infecção ativa, neoplasia maligna ou dados laboratoriais incompletos foram excluídos. Pacientes em uso de anticoagulantes, que podem afetar a função plaquetária, não foram incluídos no estudo. Pacientes hospitalizados com diagnóstico de síndrome coronariana aguda também foram excluídos. Pacientes com teste de esforço positivo e sintomas como angina de esforço foram incluídos no procedimento de angiografia. O número de pacientes com diabetes que iniciaram o uso de ácido acetilsalicílico foi semelhante em ambos os grupos. A maioria dos pacientes com ectasia coronária apresentava lesões ateroscleróticas insignificantes, causando 50% menos estenose. No entanto, nenhum dos pacientes do grupo com ECE apresentava carga de placa que pudesse causar estenose moderada a grave.

Conseguimos determinar as características clínicas básicas dos pacientes consultando o banco de dados do nosso hospital. Amostras de sangue em jejum foram coletadas durante a internação de cada paciente. Os valores bioquímicos e o hemograma completo foram realizados utilizando equipamentos automatizados. As amostras de sangue foram coletadas após jejum noturno. O escore EASIX foi calculado utilizando a seguinte fórmula: EASIX = LDH(U/L)×Creatinina(mg/dL)/Plaquetas(por nL) .

A hipertensão foi definida como pressão arterial superior a 140/90 mmHg ou uso de medicação anti-hipertensiva. O diagnóstico de diabetes mellitus foi definido por níveis de glicose plasmática em jejum de 7,0 mmol/l (126 mg/dl), hemoglobina glicada A1c de 6,5% ou uso de medicamentos antidiabéticos. A hiperlipidemia foi definida como nível de colesterol total superior a 220 mg/dl ou uso de medicação hipolipemiante. O comitê de ética do Hospital Municipal Tekirdağ Fehmi Cumalıoglu aprovou o estudo, que foi conduzido de acordo com a Declaração de Helsinque.

### Angiografia coronária

Após consentimento informado, foi realizada angiografia coronária utilizando projeções padrão através da artéria femoral direita, com abordagem típica de Judkins. Os angiogramas coronários foram examinados por dois cardiologistas distintos, que desconheciam os dados dos pacientes. Estenose de 50% ou mais em pelo menos uma artéria coronariana principal foi considerada significativa. Estenose inferior a 50% foi considerada não crítica. O aumento do diâmetro de uma artéria coronariana para 1,5 vezes ou mais do que o segmento normal local ou, frequentemente, adjacente, é conhecido como EAC.

### Análise estatística

As análises estatísticas foram realizadas utilizando o software IBM SPSS Statistics 22.0. O teste de Kolmogorov-Smirnov foi utilizado para avaliar a normalidade da distribuição dos dados. As variáveis contínuas foram expressas como média ± desvio padrão (DP). A comparação entre variáveis contínuas foi realizada utilizando o teste-t de Student para amostras independentes. As variáveis categóricas foram comparadas utilizando o teste qui-quadrado e representadas em porcentagens. A análise da curva ROC ( *Receiver Operating Characteristic* ) foi utilizada para avaliar a capacidade discriminativa, a associação com EAC e o desempenho de classificação do EASIX para EAC. Os preditores independentes de EAC foram identificados por meio de análise de regressão logística univariada e multivariada. Na análise de regressão logística, incluímos fatores que causaram EAC em estudos anteriores e dados que mostraram diferenças estatisticamente significativas entre os dois grupos. Um teste de correlação ponto-bisserial foi usado para analisar a relação entre a escore EASIX e a EAC. Um valor de p < 0,05 foi considerado estatisticamente significativo.

## Resultados

As características basais e os resultados laboratoriais dos pacientes estão resumidos na Tabela 1 . A idade média dos 300 pacientes era de 58±9 anos, sendo 53% do sexo masculino. Os dois grupos eram semelhantes em termos de características demográficas, como hiperlipidemia, hipertensão, tabagismo e diabetes mellitus.

**Tabela 1:** – Características basais dos grupos

Variável	Grupo 1 (EAC(+)) (n=150)	Grupo 2 (EAC(-)) (n=150)	Total (n=300)	Valor de p
**Idade, em anos**	60±10	57±8	58±9	0,25
**Masculino**	81 (54%)	78 (52%)	159 (53%)	0,82
**Altura, m**	1,7±0,69	1,7±0,80	1,7±0,73	0,80
**Hiperlipidemia**	45 (30%)	35 (23,3%)	80 (26%)	0,07
**Hipertensão**	39 (26%)	44 (29,3%)	83 (27,6%)	0,65
**Fumante**	37 (24,6%)	31 (20,6%)	68 (22,6%)	0,45
**Diabetes mellitus**	42 (28%)	38 (25,3%)	80 (26,6%)	0,79
**Tratamento farmacológico**				
Bloqueador de canal de cálcio	12 (8%)	11 (7,3%)	23 (7,6%)	0,79
iECA	30 (20%)	24 (16%)	54 (18%)	0,56
Ácido acetilsalicílico	38 (25,3%)	36 (24%)	74 (24,6%)	0,96
Antidiabético oral	29 (19,3%)	20 (13,3%)	49 (16,3%)	0,06
Estatina	29 (19,3%)	27 (18%)	56 (18,6%)	0,95
**Parâmetros de laboratório**				
Glicose, mg/dl	152 ±75	123 ±41	139 ±63	0,07
Hemoglobina, g/dl	14,4±3,5	14±1,6	14,2±2,7	0,87
Creatinina, mg/dl	0,96±0,25	0,93±0,43	0,95 ±0,3	0,71
Colesterol total, mg/dl	213±50	205±42	209±30	0,44
Triglicerídeos, mg/dl	173±88	165±77	169±108	0,44
Contagem de glóbulos brancos (×103/µl)	9,0±7,4	8,1±2,3	8,6±5,6	0,21
Contagem de plaquetas (×103/µl)	206±57	258±55	230±61	0,06
LDH (U/L)	300±61,4	277±69,3	289±65,5	0,96
Escore EASIX	1,46 ± 0,33	0,98 ± 0,74	1,22±2,3	< 0,001

Os dados que seguem uma distribuição normal são apresentados como média ± desvio padrão. iECA: Inibidores da enzima conversora de angiotensina; LDH: desidrogenase láctica; EAC: ectasia da artéria coronariana; EASIX: índice de ativação e estresse endotelial.

Também não houve diferença entre os dois grupos em termos de tratamento médico dos pacientes incluídos no estudo.

A contagem de PLT, LDH, hemoglobina e níveis de glicose foram semelhantes em ambos os grupos.

A escore EASIX foi maior no grupo 1. Quando o valor de corte do escore EASIX foi ≥0,74, a análise ROC apresentou poder preditivo moderado para prever EAC ( Figura 1 ).

**Figura 1:**
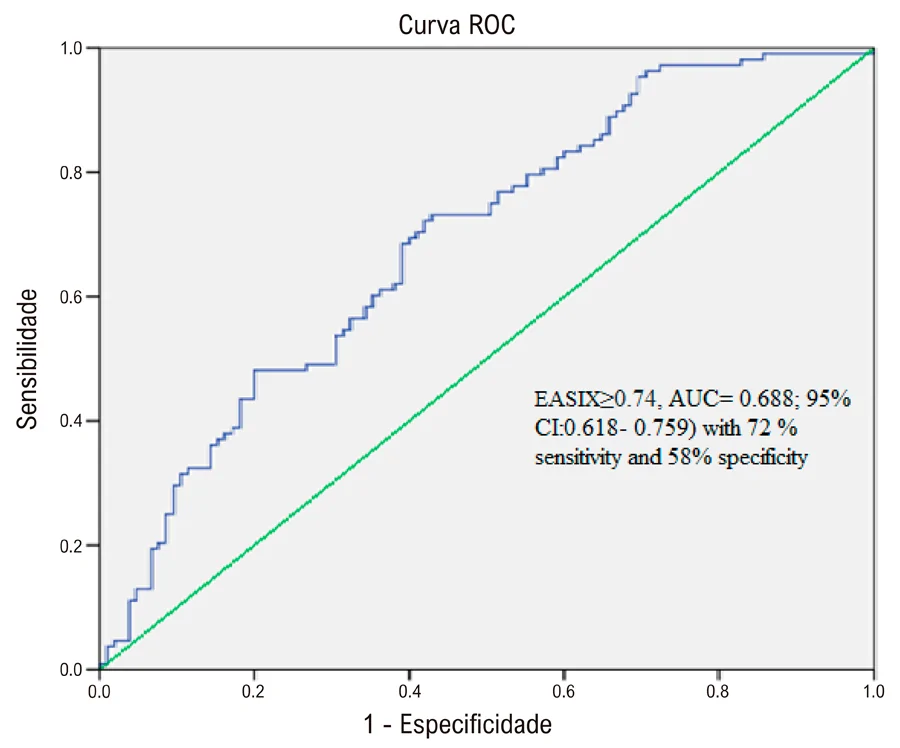
– Desempenho diagnóstico do escore índice de ativação e estresse endotelial na ectasia da artéria coronariana.

Na análise de regressão logística univariada e multivariada, um escore EASIX elevado foi um preditor independente de EAC ( Tabela 2 ).

**Tabela 2:** – Análise de regressão logística univariada e multivariada das variáveis independentes de ectasia da artéria coronariana

	Análise univariada		Análise multivariável	
Variáveis	OR (IC 95%)	Valor de p	OR (IC 95%)	Valor de p
Escore EASIX	1,3 (1,24-1,480)	0,002	2,12 (1,35–3,34)	0,001
Hipertensão	1,54 (0,895-3,427)	0,119	-	-
Fumar	1,67 (0,92–3,01)	0,08	-	-
Diabetes mellitus	1,18 (0,63-2,21)	0,59	-	-
Idade	1,02 (0,998-1,048)	0,122	-	-
Sexo (masculino)	1,34 (0,67-2,53)	0,43	-	-

EASIX: índice de ativação e estresse endotelial.

Foi descoberta uma correlação moderadamente positiva entre o escore EASIX e a EAC (r=0,44). Uma correlação moderadamente negativa foi observada entre a contagem de PLT e a EAC (r=-0,48) ( Tabela 3 ). Quando os pacientes foram divididos em tercis de acordo com os valores do escore EASIX, a maior taxa de EAC foi observada no 3º tercil (taxa de 63% quando o escore EASIX >1,03). A taxa de EAC foi de 22% quando o escore EASIX estava entre 0 e 0,63 no 1º tercil, enquanto foi de 41% quando o escore EASIX estava entre 0,63 e 1,03 no 2º tercil.

**Tabela 3:** – Análise de correlação entre ectasia da artéria coronariana e outros parâmetros

Variável	Correlação EAC (r)	Valor de p
EASIX	0,44	<0,001
Creatinina	0,02	0,696
LDH	0,03	0,996
Plaquetária	−0,48	<0,001

O teste de análise de correlação ponto-bisserial foi utilizado para analisar a relação entre as variáveis e o EAC. EAC: ectasia da artéria coronariana; EASIX: índice de ativação e estresse endotelial. LDH: desidrogenase láctica.

## Discussão

O presente estudo demonstrou que o escore EASIX é significativamente maior em pacientes com EAC, sugerindo uma possível relação entre disfunção endotelial, estresse inflamatório sistêmico e o desenvolvimento dessa anomalia vascular. Até o momento, existem poucas evidências sobre biomarcadores validados especificamente para EAC. Nessa perspectiva, nossos achados podem fornecer novos dados que apoiam o uso do EASIX como um biomarcador clinicamente valioso de dano endotelial na EAC. Essa descoberta sugere que a disfunção endotelial e o estresse inflamatório sistêmico podem desempenhar um papel importante na patogênese da EAC. Até onde sabemos, este é um dos primeiros estudos a avaliar a relação entre o EASIX e a EAC.

A EAC é caracterizada pela dilatação anormal das artérias coronárias e está frequentemente associada a processos inflamatórios, disfunção endotelial e degradação da matriz extracelular vascular.^3^ Evidências crescentes sugerem que a lesão endotelial desempenha um papel central na fisiopatologia da EAC.^11 , 12^

É também sabido que a disfunção endotelial desempenha um papel crucial na homeostase vascular e na progressão de doenças. A redução da biodisponibilidade do óxido nítrico, o aumento do estresse oxidativo e um estado pró-inflamatório e pró-trombótico são consequências da disfunção endotelial. Consequentemente, os indicadores de dano endotelial podem fornecer informações cruciais sobre os mecanismos fisiopatológicos que impulsionam a EAC.

O escore EASIX integra marcadores que refletem lesão endotelial (LDH), disfunção renal (creatinina) e atividade plaquetária, tornando-se um potencial indicador de estresse endotelial sistêmico.

O escore EASIX foi originalmente desenvolvido como um biomarcador que reflete a ativação endotelial e o estresse inflamatório sistêmico. Ele integra três parâmetros laboratoriais rotineiramente medidos: LDH, creatinina sérica e contagem de PLT. Níveis elevados de LDH refletem lesão celular e inflamação, enquanto os níveis de creatinina estão associados à disfunção microvascular. A contagem de PLT, por sua vez, reflete a atividade trombótica e inflamatória. Comparada a biomarcadores individuais, a combinação desses fatores permite que o EASIX reflita de forma mais completa a natureza multifacetada do dano endotelial. O EASIX está emergindo como um importante marcador para a microangiopatia associada ao transplante após o transplante alogênico de células-tronco. Esta é uma complicação grave e frequentemente fatal, caracterizada por níveis elevados de LDH e creatinina e baixa contagem de PLT.^4^

Mais recentemente, seu valor prognóstico foi demonstrado em diversas condições cardiovasculares, incluindo DAC e hipertensão.^13^ Escores EASIX mais elevados foram associados a piores desfechos clínicos, aumento do estresse vascular e comprometimento da função endotelial em diversos contextos.^13^ Outro estudo também constatou que o EASIX foi um preditor de prevalência de AVC, mortalidade por todas as causas e morte cardiovascular relacionada a AVC.^14^

Portanto, o escore EASIX pode fornecer um marcador composto de disfunção endotelial e carga inflamatória sistêmica.

Nossos resultados corroboram a hipótese de que a disfunção endotelial contribui para o desenvolvimento da ectasia coronária. Como o EASIX é calculado utilizando parâmetros laboratoriais de rotina, ele pode servir como um marcador simples e econômico para identificar pacientes em risco.

Os valores significativamente mais elevados de EASIX observados em pacientes com EAC sugerem que esse índice pode refletir o ambiente inflamatório vascular subjacente nesses pacientes. Outro achado importante do nosso estudo é o desempenho diagnóstico moderado do EASIX na predição de EAC. A análise ROC demonstrou que o EASIX apresentou uma capacidade razoável de discriminar pacientes com EAC daqueles sem ectasia.

Estudos anteriores demonstraram que índices inflamatórios derivados de parâmetros laboratoriais de rotina podem ser úteis em doenças cardiovasculares. Células endoteliais e PLT danificadas interagem, causando agregação plaquetária circulante e ativação plaquetária, o que aumenta o consumo de PLT. O aumento de PLT reticuladas, um biomarcador estabelecido e significativamente associado à mortalidade em distúrbios cardiovasculares, pode ser usado para visualizar o desvio à esquerda na produção de PLT resultante.^15 , 16^ Pesquisas anteriores enfatizaram a importância de índices baseados em inflamação na avaliação de EAC, incluindo a razão neutrófilo-linfócito, a razão plaqueta-linfócito e índices imunoinflamatórios sistêmicos.^6^ No entanto, o dano endotelial não é imediatamente capturado por esses indicadores, que refletem principalmente a inflamação sistêmica. Por outro lado, ao incluir dados relacionados à atividade endotelial, disfunção microvascular e equilíbrio trombótico, o EASIX oferece mais informações fisiopatológicas. Seu aspecto integrativo destaca sua potencial relevância clínica e pode explicar sua relação com a EAC. Além disso, nesse contexto, a escore EASIX pode fornecer informações adicionais, refletindo o estresse endotelial além da inflamação sistêmica.

Em um estudo recente envolvendo 1283 pacientes, o escore EASIX foi identificado como um potencial biomarcador capaz de predizer óbitos em pacientes com DAC, independentemente de ter sido realizado antes ou depois do cateterismo.^9^

Outro fator conhecido na fisiopatologia da hipertensão é a disfunção endotelial subjacente.^17^ Em outro estudo, o escore EASIX demonstrou ser um indicador de estresse vascular em pacientes hipertensos. O EASIX demonstrou bom desempenho discriminativo na identificação de pacientes hipertensos com controle inadequado da pressão arterial durante a monitorização ambulatorial da pressão arterial de 24 horas.^18 , 19^

As implicações clínicas de nossas descobertas são notáveis. Identificar pacientes com aumento da ativação endotelial pode ajudar a melhorar a estratificação de risco em indivíduos com EAC. Além disso, o EASIX pode potencialmente servir como um novo biomarcador que reflete a atividade inflamatória vascular nesses pacientes ( Figura Central ).

### Limitações do estudo

Este estudo apresenta algumas limitações que devem ser consideradas. Primeiro, nossos achados podem não ser tão amplamente aplicáveis quanto poderiam ser devido ao desenho retrospectivo e unicêntrico. Segundo, o poder estatístico da análise pode ter sido afetado pelo tamanho relativamente pequeno da amostra. Terceiro, o valor prognóstico do EASIX na predição de desfechos clínicos a longo prazo não pode ser avaliado devido à ausência de dados de acompanhamento longitudinal. Além disso, não foi possível descartar completamente a possibilidade de variáveis de confusão afetando os componentes do EASIX. Portanto, estudos prospectivos multicêntricos com amostras maiores são necessários para confirmar nossos resultados e esclarecer a utilidade clínica do EASIX em pacientes com EAC.

## Conclusão

Pacientes com EAC apresentaram níveis consideravelmente mais elevados de EASIX, que também foram associados independentemente à condição. Esses resultados corroboram a teoria de que a fisiopatologia da EAC pode envolver mecanismos relacionados à inflamação e ao estresse endotelial. Para validar esses resultados e esclarecer os mecanismos moleculares subjacentes, são necessárias mais pesquisas prospectivas.
