# Supplementary material for: Relação entre Ativação Endotelial e Índice de Estresse e Ectasia da Artéria Coronariana
Source: Arq Bras Cardiol. 2026 Sep 21;123(9):e20260242. [Article in Portuguese] doi: 10.36660/abc.20260242 (PMC13671129; doi:10.36660/abc.20260242)
Supplement: Translation

# Translation: Relationship between Endothelial Activation and Stress Index and Coronary Artery Ectasia

**DOI:** 10.36660/abc.20260242i

**Authors:** Cihan Aydin, Hüseyin Orta, Aykut Demirkiran, Nadir Emlek

**Affiliations:** 1 Türkiye Department of Cardiology, Faculty of Medicine, Tekirdag Namık Kemal University, Tekirdag – Türkiye; 2 Türkiye Department of Cardiology, Department of Cardiology, Faculty of Medicine Recep Tayyip Erdoğan University, Rize – Türkiye

**Keywords:** Vascular Endothelium, Pathologic Dilatation, Biomarkers

## Abstract

**Background:**

Coronary artery ectasia (CAE) is characterized by abnormal dilatation of the coronary arteries and is associated with endothelial dysfunction and systemic inflammation. The Endothelial Activation and Stress Index (EASIX) has emerged as a novel biomarker reflecting endothelial activation and inflammatory stress.

**Objectives:**

This study aimed to evaluate the association between EASIX score and the presence of CAE.

**Methods:**

A total of 300 patients who underwent coronary angiography between July 2018 and December 2025 were retrospectively analyzed. The study was designed as a case-control study. Patients were divided into two groups according to the presence of CAE (150 CAE, 150 controls). The EASIX score was calculated using the following formula: EASIX=LDH × Creatinine/Platelet . Receiver operating characteristic (ROC) analysis was performed to determine the predictive value of EASIX for CAE. Youden index statistical analysis was performed to evaluate the diagnostic power of ROC analysis and to calculate the optimal cut-off value. A p-value < 0.05 was considered statistically significant.

**Results:**

Patients with CAE had significantly higher EASIX score compared with controls (1.46 ± 0.33 vs. 0.98 ± 0.74). In the ROC analysis, EASIX score had 72% sensitivity and 58% specificity when the cut-off value for EASIX≥ 0.74 (AUC=0.688, 95% CI:0.618-0.759, p<0.001). In Multivariable logistic regression analysis, the EASIX score was identified as an independent predictor of CAE (OR =2.12, 95% CI: 1.35–3.34, p=0.001).

**Conclusion:**

The EASIX score was independently associated with the presence of CAE and demonstrated moderate discriminative ability.

## Introduction

Coronary artery ectasia (CAE) is defined as localized or diffuse dilation of a coronary artery exceeding 1.5 times the diameter of adjacent normal segments.^1^Ectasia refers to coronary artery enlargements up to 1.5 times, whereas aneurysms refer to enlargements more than two times.^2^CAE can be acquired or congenital. Despite being frequently regarded as a variation of coronary artery disease (CAD), CAE is a separate clinical entity with different hemodynamic and pathophysiological characteristics. The clinical significance of CAE in routine cardiology practice is shown by reports that its incidence in patients having coronary angiography ranges from 1% to 5%.^3^

Atherosclerosis accounts for 50% of the pathogenesis, while congenital syndromes such as Kawasaki syndrome, Ehlers-Danlos syndrome, Marfan syndrome and Takayasu disease, or inflammatory connective tissue diseases such as scleroderma, polyarteritis nodosa and systemic lupus erythematosus are also involved.^3^These various etiological factors imply that CAE is a complicated vascular disease involving several biological pathways rather than just a morphological anomaly.

Although the exact pathophysiology remains unclear, inflammation, endothelial dysfunction, and degradation of the extracellular matrix are considered major mechanisms. Specifically, excessive activation of proteolytic enzymes such as matrix metalloproteinases can damage the arterial wall and cause inappropriate dilatation. Endothelial dysfunction is a key factor in the development of vascular problems. Endothelial dysfunction increases thrombogenicity, impairs vascular tone regulation, and modifies coronary blood flow dynamics, all of which may put patients at risk for unfavorable cardiovascular events.

Recently, the Endothelial Activation and Stress Index (EASIX), which is calculated using standard lab tests, has been introduced as a new way to measure endothelial damage and inflammatory stress. EASIX was first used in hematological settings, but it has since been demonstrated to be linked to a poor prognosis in a variety of systemic and cardiovascular disorders. Lactic dehydrogenase (LDH), creatinine, and platelets (PLT) are included in the EASIX, which was recently developed and validated to measure the extent of endothelial damage following allogeneic stem-cell transplantation.^4^The relationship between EASIX and several endothelial activation markers has been clarified: Tumorigenicity-2 suppressor, interleukin-18 (IL-18), chemokine-X-C-ligand 8 (CXCL8), and insulin-like growth factor-1.^5^LDH, creatinine, and platelet count together represent tissue damage, renal function, and hemostatic balance, all of which are intimately related to endothelial integrity.^4 , 6^Additionally, in a number of populations, including patients with COVID-19, multiple myeloma, small-cell lung cancer, diffuse large B-cell lymphoma, sepsis, and CAD, EASIX is a valid indication of endothelial dysfunction and mortality risk.^7 - 10^

EASIX score has been associated with adverse outcomes in several cardiovascular and systemic diseases.^6^Several studies have demonstrated its utility in predicting mortality among patients with CAD, acute myocardial infarction, stroke, and hypertension.^4 , 6^However, existing data on the association between EASIX score and CAE are limited and inconclusive.

Therefore, this study aimed to evaluate the association between EASIX score and the presence of CAE, and to explore the potential clinical relevance of EASIX in this population.

## Methods

### Study population

Between July 2018 and December 2025, 150 patients who underwent angiography at our clinic and were diagnosed with CAE, and 150 patients with normal coronary arteries who were detected during the angiography procedure were retrospectively and randomly included in the study as a control group. We designed our study as a retrospective case-control study.

CAE was defined as the coronary artery lumen exceeding the maximum diameter of an adjacent normal vessel by more than 1.5 times.^1^Significant occlusion and CAD were defined as having more than 50% coronary artery stenosis. Patients with severe systemic disease, active infection, malignancy, or missing laboratory data were excluded. Patients taking anticoagulants, which can affect platelet function, were not included in the study. Patients hospitalized with a diagnosis of acute coronary syndrome were not included in the study. Patients with a positive stress test and symptoms such as exertional angina were included in the angiography procedure. The number of patients with diabetes who had been started on acetylsalicylic acid was similar in both groups. Most patients with coronary ectasia had insignificant atherosclerotic lesions causing 50% less stenosis. However, none of the patients in the CAE group had a plaque burden that would cause moderate to severe stenosis.

We were able to ascertain the patients’ basic clinical characteristics by looking through our hospital’s database. Fasting blood samples were obtained during each patient’s hospital stay. Biochemical values and whole blood counts were performed on the blood using automated equipment. Blood samples were obtained after overnight fasting. The EASIX score was calculated using the following formula: EASIX = LDH (U/L) × Creatinine (mg/dL)/ Platelet (per nL) .

Hypertension was defined as having a blood pressure of more than 140/90 mmHg or using an antihypertensive medication. Diabetes mellitus was thought to be indicated by fasting plasma glucose levels of 7.0 mmol/l (126 mg/dl), glycated haemoglobin A1c of 6.5%, or usage of antidiabetic drugs. Hyperlipidaemia was defined as having a total cholesterol level more than 220 mg/dl or being on lipid-lowering medication. The ethical committee at Tekirdağ Fehmi Cumalıoglu City Hospital accepted the study, which was carried out in accordance with the Helsinki Declaration.

### Coronary angiography

Following informed consent, coronary angiography was performed using standard projections via the right femoral artery using a typical Judkins’ approach. Coronary angiograms were examined by two separate cardiologists who were blind to the patient data. 50% or greater in at least one major coronary artery was considered significant stenosis. Less than 50% was considered non-critical coronary artery stenosis. The enlargement of a coronary artery to 1.5 times or more that of the local or often adjacent normal coronary artery segment is known as CAE.

### Statistical analysis

Statistical analyses were performed using IBM SPSS 22.0 Statistics. The Kolmogorov-Smirnov test was used to assess the normality of data distributions. Continuous variables were expressed as mean ± standard deviation (SD). Continuous variables were compared using the independent samples t-test. Categorical variables were compared using the chi-square test and represented as percentages. Receiver operating characteristic (ROC) analysis was used to discriminative ability, association with CAE, or classification performance of EASIX for CAE. CAE’s independent predictors were found using univariate and Multivariable logistic regression analysis. In the logistic regression analysis, we included factors that caused CAE in previous studies and data showing statistically significant differences between the two groups. A point-biserial correlation analysis test was used to analyze the relationship between the EASIX score and CAE. A p-value < 0.05 was considered statistically significant.

## Results

The baseline characteristics and laboratory results of the patients are summarized in Table 1 . The 300 patients’ average age was 58±9 years, and 53 % of them were male. The two groups were similar in terms of demographic characteristics such as hyperlipidemia, hypertension, smoking, and diabetes mellitus.

**Table 1:** – Baseline characteristics of the groups

Variable	Group 1 (CAE(+)) (n=150)	Group 2 (CAE(-)) (n=150)	Total (n=300)	p - value
**Age, yrs**	60±10	57±8	58±9	0.25
**Male**	81 (54%)	78 (52%)	159 (53%)	0.82
**Height, m**	1.7±0.69	1.7±0.80	1.7±0.73	0.80
**Hyperlipidemia**	45(30%)	35 (23.3%)	80(26%)	0.07
**Hypertension**	39 (26%)	44 (29.3%)	83 (27.6%)	0.65
**Smoker**	37 (24.6%)	31 (20.6%)	68(22.6%)	0.45
**Diabetes mellitus**	42 (28%)	38(25.3%)	80(26.6%)	0.79
**Pharmacological Treatment**				
Ca–channel blocker	12(8%)	11(7.3%)	23(7.6%)	0.79
ACE-I	30 (20%)	24 (16%)	54 (18%)	0.56
Acetyl salicylic acid	38 (25.3%)	36(24%)	74 (24.6%)	0.96
Oral antidiabetic	29 (19.3%)	20 (13.3%)	49 (16.3%)	0.06
Statin	29 (19.3%)	27 (18%)	56 (18.6%)	0.95
**Laboratory parameters**				
Glucose, mg/dl	152 ±75	123 ±41	139 ±63	0.07
Hemoglobin, g/dl	14.4±3.5	14±1.6	14.2±2.7	0.87
Creatinine, mg/dl	0.96±0.25	0.93±0.43	0.95 ±0.3	0.71
Total cholesterol, mg/dl	213±50	205±42	209±30	0.44
Triglycerides, mg/dl	173±88	165±77	169±108	0.44
White blood cell count (×10^3^/µl)	9.0±7.4	8.1±2.3	8.6±5.6	0.21
Platelet count (×10^3^/µl)	206±57	258±55	230±61	0.06
LDH (U/L)	300±61.4	277±69.3	289±65.5	0.96
EASIX Score	1.46 ± 0.33	0.98 ± 0.74	1.22±2.3	< 0.001

Data following a normal distribution are presented as mean ± standard deviation. CAE: coronary artery ectasia; ACE-I: angiotensin-converting enzyme inhibitors; LDH: lactic dehydrogenase.

There was also no difference between the two groups in terms of medical treatment of the patients included in the study.

Platelet count, LDH, hemoglobin, and glucose levels were similar in both groups.

EASIX score was higher in group 1. When the EASIX score cut-off value was≥0.74, the ROC analysis had moderate predictive power to predict CAE. ( Figure 1 ).

**Figure 1:** – Diagnostic Performance of EASIX Score in CAE

In Univariate and Multivariable logistic regression analysis, a high EASIX score was an independent predictor of CAE ( Table 2 ).

**Table 2:** – Univariate and Multivariable logistic regression analysis of the independent variables of CAE

	Univariate analysis		Multivariable analysis	
Variables	OR(95% CI)	p - value	OR(95% CI)	p - value
EASIX score	1.3(1.24-1.480)	0.002	2.12 (1.35–3.34)	0.001
Hypertension	1.54(0.895-3.427)	0.119	-	-
Smoking	1.67(0.92–3.01)	0.08	-	-
Diabetes mellitus	1.18(0.63-2.21)	0.59	-	-
Age	1.02(0.998-1.048)	0.122	-	-
Sex(male)	1.34(0.67-2.53)	0.43	-	-

EASIX: Endothelial Activation and Stress Index.

A moderately positive correlation was discovered between the EASIX score and CAE (r=0.44). A moderately negative correlation was observed between platelet count and CAE (r=-0.48) ( Table 3 ). When patients were divided into tertiles according to EASIX score values, the highest CAE rate was observed in the 3rd tertile (CAE rate was 63% when EASIX score >1.03). The CAE ratio was 22% when the EASIX score was between 0-0.63 in tertile 1, while it was 41% when the EASIX score was between 0.63-1.03 in tertile 2.

**Table 3:** – Correlation Analysis of CAE and other parameters.

Variable	CAE correlation (r)	p - value
EASIX	0.44	<0.001
Creatinine	0.02	0.696
LDH	0.03	0.996
Platelet	−0.48	<0.001

Point-biserial correlation analysis test was used to analyze the relationship between the variables and the CAE: coronary artery ectasia; EASIX: Endothelial Activation and Stress Index; LDH; lactic dehydrogenase.

## Discussion

The present study demonstrated that the EASIX score is significantly higher in patients with CAE, suggesting a potential relationship between endothelial dysfunction, systemic inflammatory stress, and the development of this vascular anomaly. To date, there is limited evidence regarding validated biomarkers specifically for CAE. In this perspective, our findings may provide new data supporting the use of EASIX as a clinically valuable biomarker of endothelial damage in CAE. This finding suggests that endothelial dysfunction and systemic inflammatory stress may play an important role in the pathogenesis of CAE. To the best of our knowledge, this is among the first studies evaluating the relationship between EASIX and CAE.

CAE is characterized by abnormal dilatation of the coronary arteries and is frequently associated with inflammatory processes, endothelial dysfunction, and degradation of the vascular extracellular matrix.^3^Increasing evidence suggests that endothelial injury plays a central role in the pathophysiology of CAE.^11 , 12^

It is also well known that endothelial dysfunction plays a crucial role in vascular homeostasis and the advancement of illness. Reduced nitric oxide bioavailability, elevated oxidative stress, and a pro-inflammatory and pro-thrombotic state are all consequences of endothelial dysfunction. Consequently, endothelial damage indicators may offer crucial information on the pathophysiological mechanisms driving CAE.

The EASIX score integrates markers reflecting endothelial injury (LDH), renal dysfunction (creatinine), and platelet activity, making it a potential indicator of systemic endothelial stress.

The EASIX score was originally developed as a biomarker reflecting endothelial activation and systemic inflammatory stress. It integrates three routinely measured laboratory parameters: lactate dehydrogenase, serum creatinine, and platelet count. Elevated LDH levels reflect cellular injury and inflammation, while creatinine levels are associated with microvascular dysfunction. Platelet count, on the other hand, reflects thrombotic and inflammatory activity. Compared to individual biomarkers, the combination of these factors enables EASIX to more fully reflect the multifaceted nature of endothelial damage. EASIX is emerging as an important marker for transplant-associated microangiopathy following allogeneic stem cell transplantation. This is a serious and often fatal complication characterized by elevated LDH and creatinine levels and a low platelet count.^4^

More recently, its prognostic value has been demonstrated in various cardiovascular conditions, including CAD and hypertension.^13^Higher EASIX scores have been linked to worse clinical outcomes, elevated vascular stress, and compromised endothelial function in various contexts.^13^Another study also found that EASIX was a predictor of stroke prevalence, all-cause mortality, and stroke-related cardiovascular death.^14^

Therefore, the EASIX score may provide a composite marker of endothelial dysfunction and systemic inflammatory burden.

Our findings support the hypothesis that endothelial dysfunction contributes to the development of coronary ectasia. Because EASIX is calculated using routine laboratory parameters, it may serve as a simple and cost-effective marker for identifying patients at risk.

The significantly higher EASIX values observed in patients with CAE suggest that this index may reflect the underlying vascular inflammatory milieu in these patients. Another important finding of our study is the moderate diagnostic performance of EASIX in predicting CAE. The ROC analysis demonstrated that EASIX had a reasonable ability to discriminate patients with CAE from those without ectasia.

Previous studies have shown that inflammatory indices derived from routine laboratory parameters may be useful in cardiovascular diseases. Damaged endothelial cells and PLT interact to cause circulating platelet aggregates and platelet activation, which raises platelet consumption. Increased reticulated PLT, an established biomarker significantly linked to mortality in cardiovascular disorders, can be used to visualise the resulting left-shifted platelet production.^15 , 16^Prior research has emphasized the importance of inflammation-based indices in the evaluation of CAE, including neutrophil-to-lymphocyte ratios, platelet-to-lymphocyte ratios, and systemic immune-inflammatory indices.^6^However, endothelial damage is not immediately captured by these indicators, which mainly reflect systemic inflammation. On the other hand, by including data related to endothelial activity, microvascular dysfunction, and thrombotic balance, EASIX offers more pathophysiological information. Its integrative aspect highlights its potential clinical relevance and may account for its relationship with CAE. Furthermore, in this context, the EASIX score can provide additional information by reflecting endothelial stress in addition to systemic inflammation.

In a recent study involving 1283 patients, the EASIX score was identified as a potential biomarker that could predict death in patients with CAD, regardless of whether it was performed before or after catheterization.^9^

Another known factor in the pathophysiology of hypertension is underlying endothelial dysfunction.^17^In another study, the EASIX score was shown to be an indicator of vascular stress in hypertensive patients. EASIX demonstrated good discriminative performance in identifying hypertensive patients with inadequate blood pressure control during 24-hour ambulatory BP monitoring.^18 , 19^

The clinical implications of our findings are noteworthy. Identifying patients with increased endothelial activation may help improve risk stratification in individuals with CAE. Moreover, EASIX could potentially serve as a novel biomarker reflecting vascular inflammatory activity in these patients ( Central Illustration ).

### Study limitations

This study has a number of limitations that should be noted. First, our findings may not be as broadly applicable as they could be due to the retrospective and single-center design. Second, the analysis’s statistical power might have been impacted by the comparatively small sample size. Third, the prognostic value of EASIX in predicting long-term clinical outcomes cannot be evaluated due to the absence of longitudinal follow-up data. Furthermore, it was not possible to completely rule out the possibility of confounding variables affecting EASIX components. Larger, prospective, multicenter studies are therefore necessary to confirm our results and clarify the clinical usefulness of EASIX in CAE patients.

## Conclusion

Patients with CAE had considerably higher EASIX levels, which were also independently associated with the condition. These results lend credence to the theory that the pathophysiology of CAE may involve mechanisms related to inflammation and endothelial stress. To validate these results and clarify the underlying molecular mechanisms, more prospective research is required.
